# Supplementary material for: Molecular Mechanisms of the Stripe Rust Interaction with Resistant and Susceptible Wheat Genotypes
Source: Int J Mol Sci. 2024 Mar 2;25(5):2930. doi: 10.3390/ijms25052930 (PMC10931583; doi:10.3390/ijms25052930)
Supplement: Supplementary file 1 [file ijms-25-02930-s001.zip › ijms-2877432-supplementary.pdf]

Supplementary Table S1. Information of mapping statistics for all the transcriptome reads to *Pst*-78 reference genome.

	**Resistant bulk (count)**	**Susceptible bulk (count)**	**Resistant (%)**	**Susceptible (%)**
**Counted fragments**	370,937	352,479	10.18	11.91
**Unique fragments**	342,405	329,772	9.40	11.15
**Common fragments**	28,532	22,707	0.78	0.77
**Uncounted fragments**	3,271,563	2,606,435	89.82	88.09
**Total fragments**	3,642,500	2,958,914	100	100

Note: Counted fragments represent reads that mapped to reference *Pst* genome, unique fragments mapped to single position on the genome, while non-specific (aligned to a few regions) had between 1-5 hits, uncounted fragments didn't have any hits in the reference *Pst* genome.
